# Lung transplantation with concomitant cardiac repair for congenital hypoplasia of bilateral pulmonary arteries and patent ductus arteriosus

**DOI:** 10.1186/s13019-022-01792-z

**Published:** 2022-03-23

**Authors:** Che-Chih Cheng, Ming-Tai Lin, Shu-Chien Huang, Hsao-Hsun Hsu

**Affiliations:** 1 Department of Surgery, Kaohsiung Armed Forces General Hospital, Kaohsiung, Taiwan; 2 Division of Thoracic Surgery, Department of Surgery, National Taiwan University Hospital and National Taiwan University College of Medicine, Taipei, Taiwan; 3 Department of Pediatrics, National Taiwan University Hospital, Taipei, Taiwan; 4 College of Medicine, National Taiwan University, Taipei, Taiwan

**Keywords:** Congenital heart disease, Lung transplantation, Patent ductus arteriosus

## Abstract

**Background:**

Profound pulmonary arterial hypertension with end-stage right heart failure is considered to be the main cause of death in children with un-repaired congenital heart disease, and the traditional surgical treatment is heart–lung transplantation. We performed bilateral lung transplantation (LTx) with concomitant cardiac repair, and the patient has uplifting outcome.

**Case presentation:**

We have reported the case of a patient with congenital hypoplasia of the bilateral pulmonary arteries and patent ductus arteriosus. The patient’s clinical condition was gradually worsening and severely limited his ability to perform the activities of daily life. Bilateral LTx with concomitant patent ductus arteriosus repair was performed at the age of 11 years. The postoperative course was smooth and cardiopulmonary function nearly returned to normal according to radiological and laboratory examinations.

**Conclusions:**

Bilateral LTx with concomitant cardiac repair may be superior to heart–lung transplantation in the case of the specific congenital heart disease.

## Background

Pulmonary hypoplasia, a rare congenital heart disease (CHD), is characterized by the underdevelopment of the pulmonary structure, including the lung parenchyma, bronchus, and blood vessels [1, 2]. Although the true pathogenesis is unclear, it may be associated with an accident in utero or an embryologic defect of the lung or vascular tissue, which resulted in unilateral or bilateral absence of the pulmonary artery. For patients with congenital hypoplasia of the bilateral pulmonary arteries, the pulmonary circulation depends on support from the systemic collateral circulation. The lack of normal pulmonary vessels leads to erythrocytosis, chronic hypoxemia, and cyanotic multisystemic complications, which result in an increase in pulmonary vascular resistance and progressive life-threatening pulmonary arterial hypertension (PAH) [3].

We herein report the case of an 11-year-old child with congenital hypoplasia of the bilateral pulmonary arteries and patent ductus arteriosus (PDA). The patient presented with central cyanosis and consequent PAH, and received a bilateral lung transplantation (LTx) with intraoperative PDA repair for his end-stage pulmonary hypoplasia accompanied with severe PAH and right heart decompensation.

## Case presentation

**Fig. 1:**
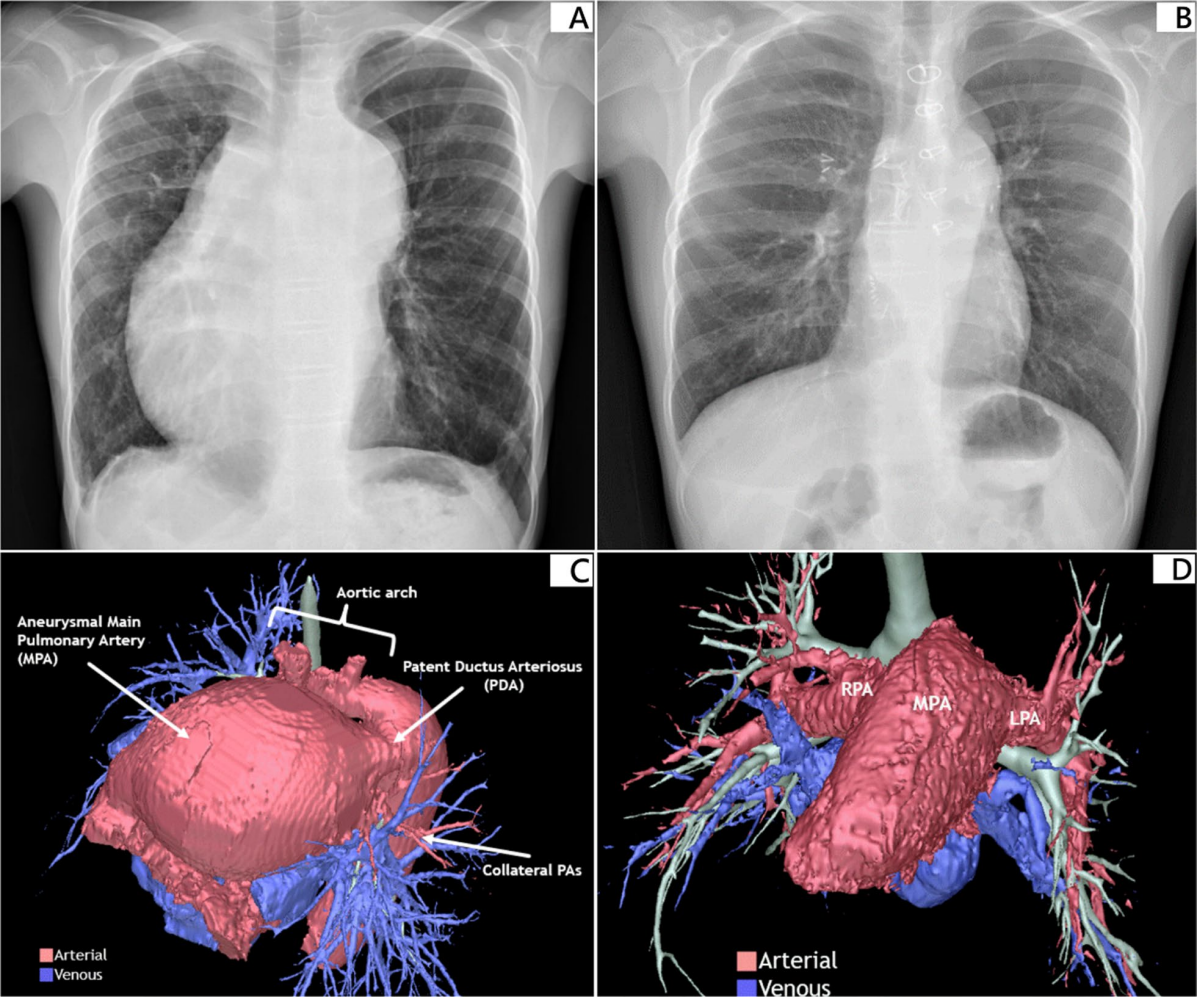
Chest radiograph (CXR) and three-dimensional reconstructed chest computed tomography (3DrCT) before and after lung transplantation. **A **CXR shows engorged main pulmonary artery and right-side cardiomegaly before lung transplantation. **B **CXR shows normal heart size with good expansion of the bilateral lung field one year after lung transplantation. **C **Preoperative 3DrCT provides clear visualization of the cardiac anatomy. Hypoplasia of the bilateral pulmonary artery with some small collateral vessels from systemic circulation (pink color), aneurysmal dilatation of the main pulmonary trunk, and a patent ductus arteriosus are well demonstrated in the reconstructive photography. **D **Postoperative 3DrCT shows the virtual images of bronchial trees and the pulmonary vessels 11 months after transplantation (pink color indicates pulmonary arterial system and blue color shows pulmonary venous system)

**Fig. 2:**
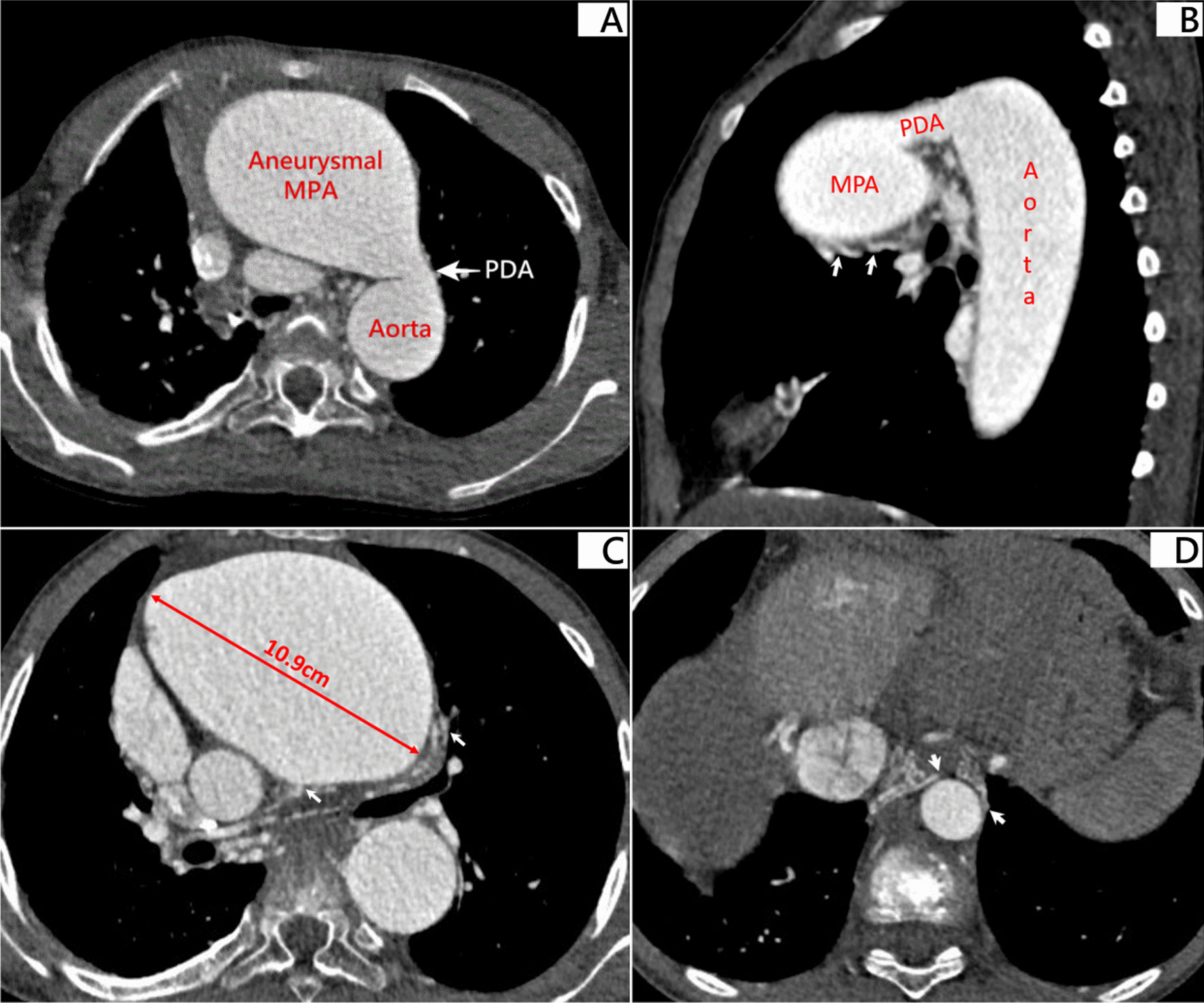
Contrast thoracic computed tomography shows the patient’s cardiothoracic anatomy before lung transplantation. **A **Patent ductus arteriosus (approximately 1.1 cm in diameter) identified between the aneurysmal main pulmonary artery and the descending aorta. **B **Axial view of thoracic computed tomography reveals patent ductus arteriosus between the main pulmonary artery and the descending aorta. Arrows indicate several collateral branches of bilateral pulmonary arteries (approximately 4–5 mm in diameter). **C **Maximum diameter of the aneurysmal main pulmonary trunk is 10.9 cm. The Arrows indicate several collateral branches of bilateral pulmonary arteries (approximately 4–5 mm in diameter). **D **Arrows indicate bilateral major aortopulmonary collateral arteries (MAPCAs) from the descending aorta (approximately 2–4 mm in diameter)

A 10-year-old boy with CHD presenting with central cyanosis and severe PAH was referred to us for LTx evaluation. The diagnosis of congenital hypoplasia of the bilateral pulmonary arteries and PDA was confirmed by right heart catheterization at birth. The PAH-targeted therapy, Sildenafil, was initially prescribed at the age of 8 weeks. In order to relieve his symptoms, the PAH combination therapy of Sildenafil and Bosentan was started at 17 weeks. The dose was modified according to his body weight. However, his clinical condition deteriorated resulting in an increasing number of hospital admissions due to end-stage CHD presenting as severe hypoxia and shortness of breath. The repeated right heart catheterization before referral showed a mean pulmonary arterial pressure of 97 mmHg (the same level as that in the ascending aorta) and the 6-min-walk test distance was only 80 m. Pre-operative chest radiography and multidetector thoracic computed tomography (CT) illustrated aneurysmal dilatation of the main pulmonary trunk and right-side cardiomegaly, accompanied with severe hypoplasia of the bilateral pulmonary arteries and a large PDA (Figs. 1A, C, and 2). After multidisciplinary evaluation, bilateral LTx under cardiopulmonary bypass support with concomitant intraoperative PDA repair was offered. The patient was then listed as status 1 exception in the LTx waiting list.

Due to extreme scarcity of cadaveric donors in Taiwan and the small size of our recipient (160 cm in height and 38 kg in weight), a suitable donor was available a year later (a brain-dead 32-year-old male with a good size match between the donor and recipient). Under precise donor-recipient team cooperation, a standard donor procurement technique with Perfadex® solution for ante-grade and retro-grade organ perfusion and preservation was performed. After median sternotomy, the PDA was resected, and the aortic side of the PDA was repaired by continued suture after cardiopulmonary bypass had been established. The oversized main pulmonary trunk was trimmed to approximately half of its original diameter using linear stapler and reinforced by 4–0 Prolene running suture. The recipient’s right lung was removed and then the donor’s right lung was implanted by sequential anastomosis of right main bronchus and left atrium first. The donor’s right main pulmonary artery was anastomosed to the recipient’s main pulmonary trunk using end-to-side anastomosis. And the left donor lung was implanted using a similar technique. The total allograft ischemia time was 513 min.

**Fig. 3:**
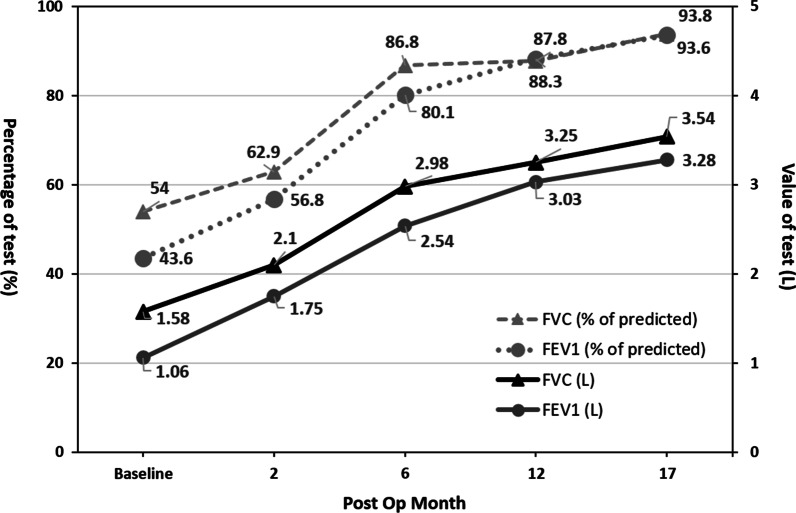
Pulmonary functional test before and after bilateral lung transplantation

**Fig. 4:**
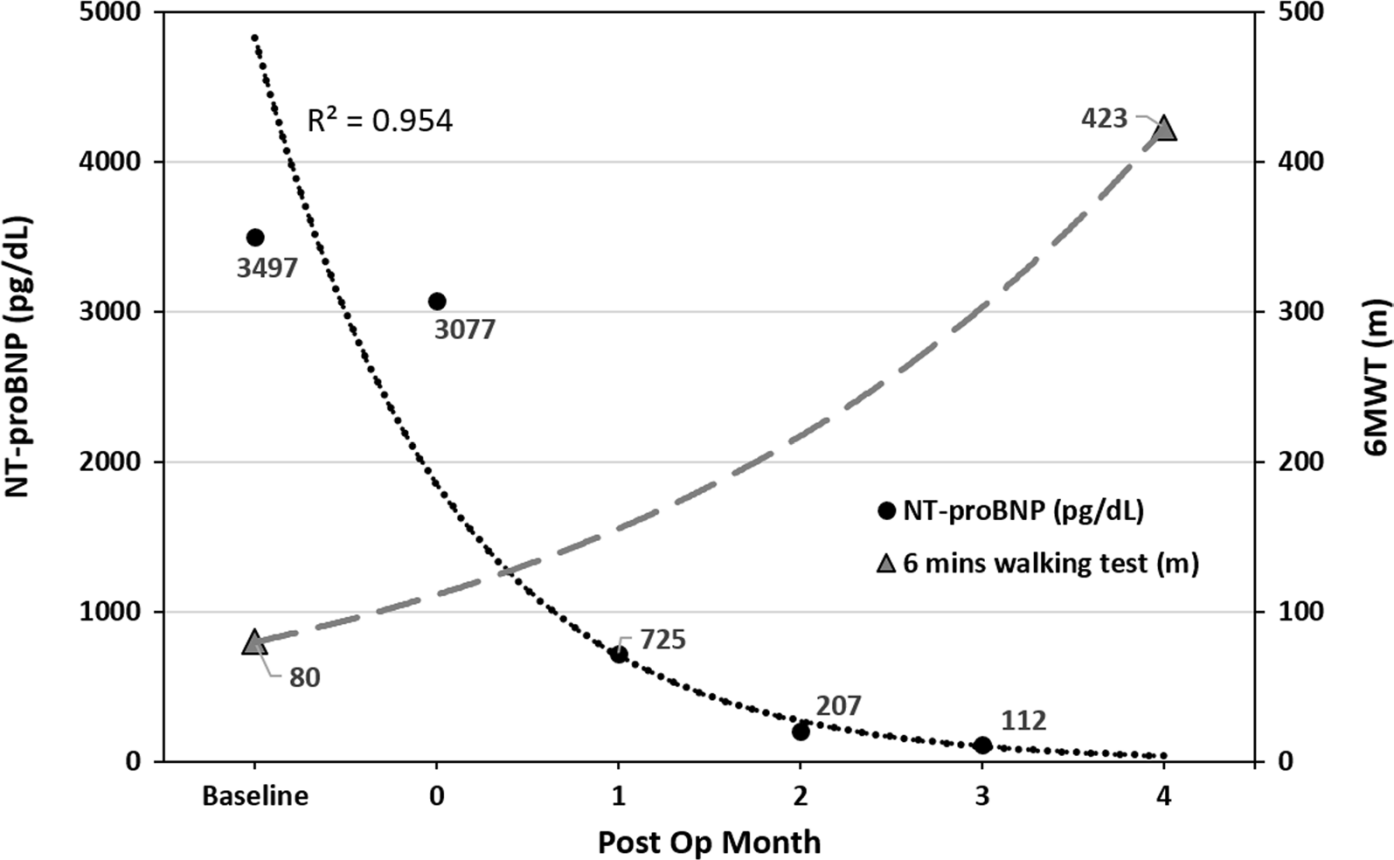
Curvilinear relationship between time and blood level of NT-proBNP and distance of 6-Minute-Walk test

The hospitalization included an intensive care unit (ICU) stay of 67 days and a general ward stay of 15 days. There were two major events during the ICU stay. The first was pneumonia with sepsis attributed to the donor’s nosocomial infection that delayed removal of the endotracheal tube; this lasted for 12 days. The second was pancytopenia with intermittent fever. After a series of examinations and drug modifications, a complex web of causes was determined, including collapsed lung pneumonia, cytomegalovirus infection related splenomegaly complicated with thrombocytopenia, and drug reaction (Tazocin), that led to an ICU stay of approximately 38 days. The patient was discharged in a relatively stable condition on postoperative day 82 after an improved clinical status and rehabilitation in the general ward. Thoracic CT revealed that the diameter of dilated pulmonary trunk significantly decreased from 10.9 cm preoperatively to 4.9 cm postoperatively. The postoperative chest radiograph demonstrated a nearly normal cardiac shadow, and a reconstructive chest CT illustrated that the patient’s anatomy was similar to normal (Fig. 1B, D). The patients exercise capacity, such as pulmonary function and 6-min-walk-test distance, improved significantly and the level of NT-pro BNP returned to the normal range, compared with that of the preoperative condition (Figs. 3, 4). The patient had no physical limitations and enjoyed a good quality of life and returned to school 1 year after surgery.

## Discussion and conclusions

Because of early diagnosis and the advances in pediatric cardiac surgery, the life expectancy of patients with CHD has improved significantly over recent decades. For CHD children with a complex anatomy who are not suitable candidates for surgical repair, unrepaired intracardiac or extracardiac shunts could lead to a progressive increase in pulmonary vascular resistance and life-threatening PAH [4, 5]. As these CHD patients grow up, a progressive decline in cardiopulmonary function due to irreversible PAH and cyanotic multisystemic complications increase the morbidities and affect their daily activities. As a consequence, most deaths in CHD patients with refractory PAH occur in adulthood. According to the “Guidelines for the Management of Grown up Congenital Heart Disease”, cardiothoracic transplantation, such as heart–lung transplantation (HLTx) or LTx with simultaneous repair of the cardiac defect, is recommended to be considered when the short-term prognosis is poor or quality of life is unacceptable [6].

For end-stage CHD patients referred for thoracic transplantation, HLTx theoretically could be accomplished with a shorter operation and bypass time because there is no need to deal with complex congenital defects and the number of anastomoses between donor and recipient is limited, compared to that of LTx with additional reconstructive surgery. For CHD patients with Eisenmenger syndrome receiving thoracic transplantation, Waddell et al. reported that the 30-day (80.7%) and 1-year survival rates (70.1%) in the HTLx group were better than those in the LTx group (68% and 55.2%) [7]. However, a cohort study conducted in Nordic patients with Eisenmenger syndrome who underwent transplantation demonstrated that there was no difference in the median survival after HLTx and LTx with concomitant repair of cardiac defects [8]. This multicenter study also illustrated that there was no survival difference between complex and simple anatomical defects, between pediatric and adult transplantations, or between centers. Compared to HLTx, the additional benefit of LTx with simultaneously cardiac repair for CHD patients is that the donor heart could be preserved and offered to another recipient on the heart transplant list. In the current era of organ scarcity, this therapeutic option would be considered favorable due to the large disparity between the patients on the waiting list and the number of cadaveric allografts available.

In conclusion, although the operation time was longer and expertise in the surgical technique is required, our case demonstrated that LTx with concomitant cardiac repair at centers with expertise in both cardiac surgery and thoracic transplantation could be an acceptable and favorable therapeutic option for some selective end-stage CHD patients with complex cardiac defects.

## Data Availability

The datasets used and/or analyzed during the current study are available from the corresponding author on reasonable request.

## Abbreviations

CHD: Congenital heart disease
CT: Computed tomography
CXR: Chest radiography
HLTx: Heart–lung transplantation
LTx: Lung transplantation
MAPCA: Major aortopulmonary collateral artery
PAH: Pulmonary arterial hypertension
PDA: Patent ductus arteriosus
3DrCT: Three-dimensional reconstructed chest computed tomography
